# WeChat as a Platform for Problem-Based Learning in a Dental Practical Clerkship: Feasibility Study

**DOI:** 10.2196/12127

**Published:** 2019-03-19

**Authors:** Wei Zhang, Zheng-Rong Li, Zhi Li

**Affiliations:** 1 State Key Laboratory Breeding Base of Basic Science of Stomatology (Hubei-MOST) and Key Lab for Oral Biomedical Engineering of the Ministry of Education School and Hospital of Stomatology Wuhan University Wuhan China; 2 Department of Endodontics School and Hospital of Stomatology Wuhan University Wuhan China; 3 Department of Oral and Maxillofacial Surgery School and Hospital of Stomatology Wuhan University Wuhan China

**Keywords:** PBL, app, WeChat, clerkship, dental medicine

## Abstract

**Background:**

Problem-Based-Learning (PBL) has been widely accepted in student-centered medical education. Since WeChat is the most popular communication app in China, we have chosen to use WeChat as new platform for online PBL in order to reduce the limitations of traditional PBL in dental practical clerkships.

**Objective:**

This study aims to demonstrate the feasibility and acceptability of online PBL using WeChat (WeChat-PBL) in a dental practical clerkship.

**Methods:**

A total of 72 students in a dental practical clerkship and 10 tutors participated in this study from June to August 2017. We created 10 WeChat groups to provide a communication platform for the PBL teaching, in which the students selected the PBL cases themselves from their practical clerkship. After each individual PBL case, group members were required to complete an evaluation on the PBL process itself. A final questionnaire survey was completed by the participants to summarize the long-term evaluation of the whole WeChat-PBL experience after the 3-month clerkship. Data from the PBL cases, WeChat messages, periodic evaluations, and long-term evaluations were collected for analysis.

**Results:**

There were 45 cases presented in the WeChat-PBL within the 3-month clerkship. All students had positive reactions to the communication within the PBL groups. The results of the periodic evaluation showed that the students and tutors were quite satisfied with the process of WeChat-PBL and appreciated the group members’ contributions and performance. The final questionnaire results indicated that the WeChat-PBL had achieved positive effects.

**Conclusions:**

The results of this study indicate the feasibility and acceptability of the app, WeChat, for problem-based learning in a dental practical clerkship.

## Introduction

Dental medicine is a practical discipline, and the clerkship is a key component of dental medicine education. The quality of the clerkship directly affects the students’ future career. In China, the dental practical clerkship is still a teacher-centered process rather than a student-centered one, leading to students who struggle to think independently and solve clinical problems by themselves. The traditional pedagogy of teacher-centered, class-oriented didactic lectures and an examination-driven curriculum put the students in a passive state of “reception” [1], which results in a lack of initiative among students in clerkships. Problem-based learning (PBL) is a widely accepted student-centered educational method focused on the discussion and learning that emerge from focusing on a clinically based problem [2,3]. As an innovative approach in medical education, the role of PBL has been well documented since the pioneering use of PBL in medical education at McMaster University (Ontario, Canada) in the late 1960s [4]. There are significant contributions from PBL in medical education, and we wish to test whether PBL can improve the quality of dental practical clerkships.

However, traditional PBL has its own limitations in a dental practical clerkship. Traditional PBL usually involves a group of 6-8 students with a tutor in a face-to-face PBL session, where the participants get together in a classroom to discuss and share opinions. The face-to-face session is essential in traditional PBL. However, due to the geographic dispersion of students, it is difficult to perform traditional PBL in a dental practical clerkship. Generally, students are assigned to different clinical departments for their clerkship rotation, which makes it hard to schedule regular meetings of the students in order to launch a PBL session. Moreover, the students and tutors lack the free time needed for PBL during busy clerkships. Therefore, modifications to traditional PBL are needed in order to get around the physical and temporal restrictions in dental clerkships.

To address these problems, we are designing a novel method to transfer traditional PBL to an online PBL setting in a virtual environment. The new mode of PBL would be built on modern digital technology. Therefore, the physical and time restrictions of traditional PBL education are eliminated, stimulating students’ autonomous learning. Mobile phones, specifically smartphones, are one of the fastest growing sectors in the Internet technology industry, and their impact in medicine has already been significant [5,6]. Many educators have used different mobile phone apps with positive results: in particular, students’ self-efficacy, self-confidence, and self-management have shown improvement [7,8]. Because mobile phone apps are easy and convenient to operate, they are becoming more and more popular in education. Social media platforms such as Facebook and Twitter have been introduced into medical education in western countries with great success [9-11].

After reflecting on the situation in China, we decided on the multipurpose messaging, social media, and mobile payment app, WeChat, as a new platform to deliver PBL to students in a dental practical clerkship. WeChat, released in 2011 by Tencent, is one of the largest standalone mobile phone messaging communication apps in China, with over 938 million global active users by 2017. In addition, WeChat has already been used as a mobile and interactive communication tool in medical education [12,13]. WeChat provides text, hold-to-talk voice and broadcast (one-to-many) messaging, instant video conferencing, and photograph or video sharing. With those functions included in WeChat, it is a suitable tool for online PBL, eliminating the physical limitations of traditional PBL.

In this study, we explored WeChat-based PBL for students in a dental practical clerkship and aimed to demonstrate its feasibility and acceptability.

## Methods

### Participants

In this study, use of the WeChat-PBL took place from June to August 2017 at the Hospital of Stomatology, Wuhan University, in China. Clerkship students practicing dental medicine in different departments (including the Departments of Oral and Maxillofacial Surgery, Endodontics, Prosthodontics, Periodontology, Orthodontics, Pediatric Dentistry, and Dental Implantology) participated in this study. During their clerkships, students are required to complete clinical rotations in different departments and perform treatments under the supervision of senior doctors. The time spent per rotation in each department is 6 weeks.

**Figure 1:**
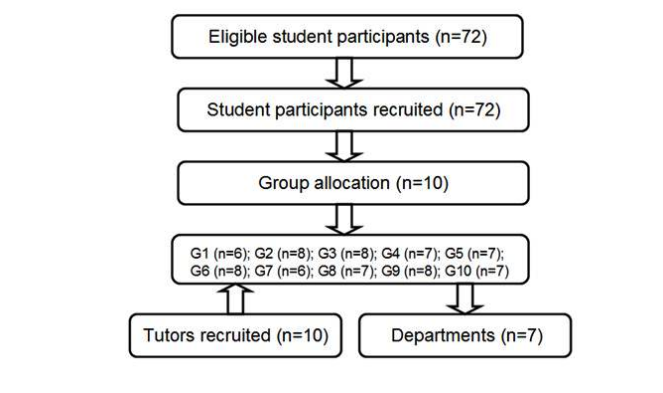
Flowchart showing the sampling process and conceptual framework.

The study was performed with dental students participating in a 3-month clerkship. Students who had traditional PBL experience in the preclinical curriculum were selected. Clinical doctors were assigned as tutors in the WeChat-PBL groups. They were required to have at least one year or more of experience in traditional PBL teaching. In total, 72 students (34 males, 38 females; mean age 23.6 years [SD 2]) and 10 tutors were included in this study (Figure 1). Informed consent was given to participants and signed by them prior to beginning the study.

All participants had their own mobile phones and were required to install WeChat. They were trained to use the practical aspects of WeChat in the PBL context. A total of 10 WeChat groups was created to provide the communication platform for the PBL teaching. The 72 students and 10 tutors from different departments were randomly assigned to each group. Six to eight students and the assigned tutor were asked to join the same WeChat group. Each group consisted of students working in different departments.

### WeChat Problem-Based Learning Mode

**Figure 2:**
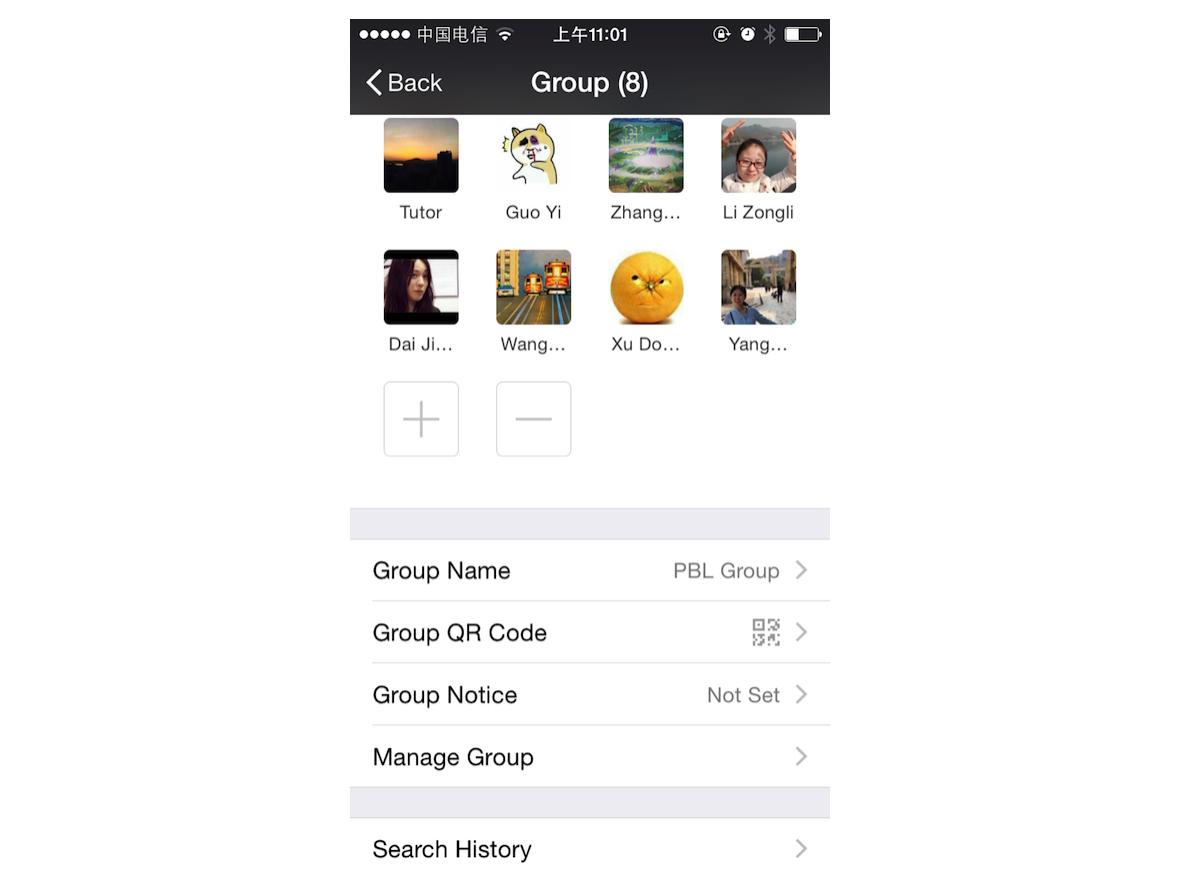
One of the problem-based-learning groups (7 students and a tutor).

**Figure 3:**
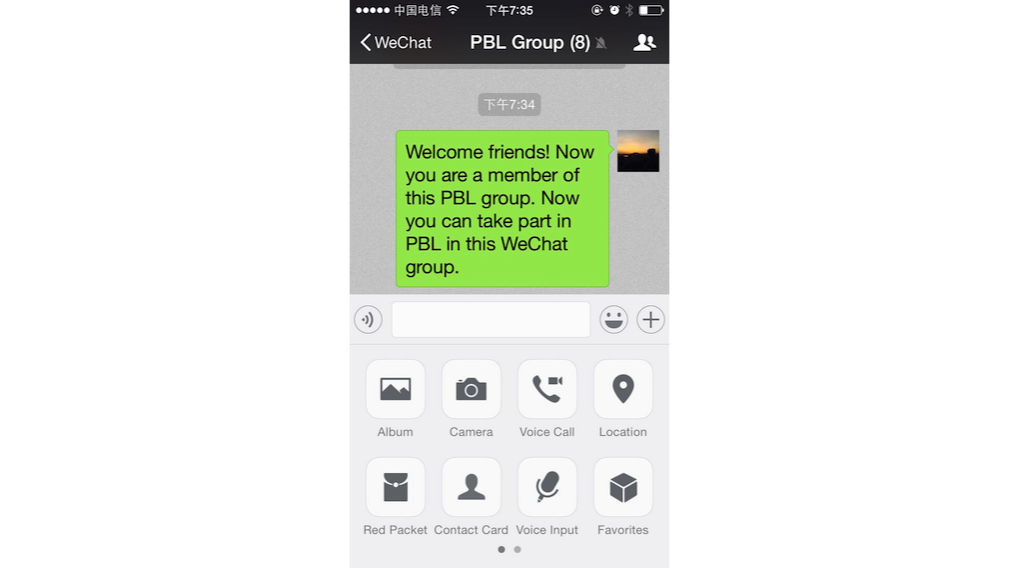
User interface for chatting, which was used to send and receive texts, images, voice messages, videos, and documents.

**Figure 4:**
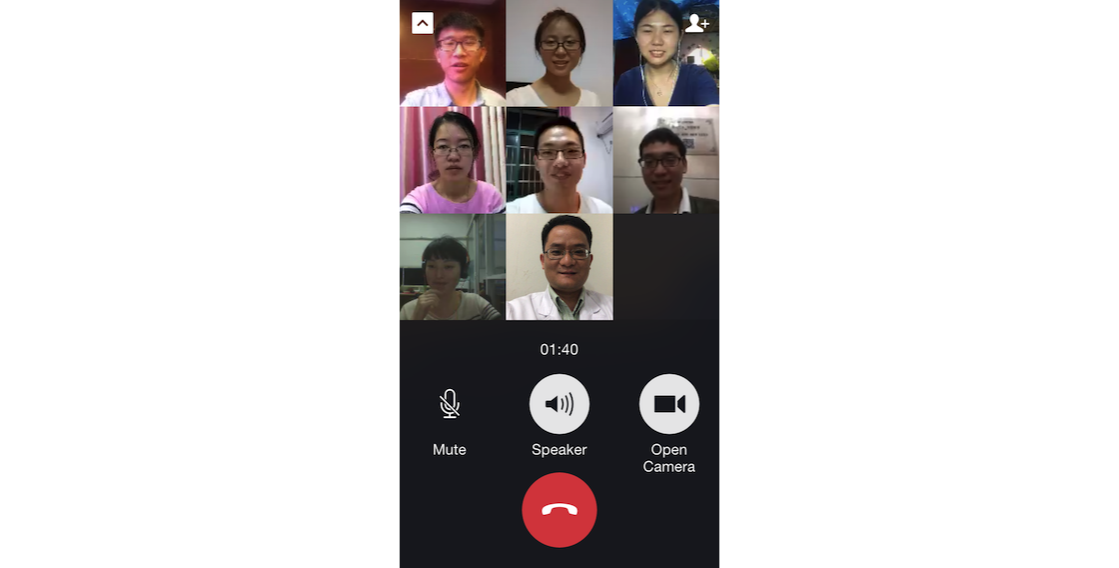
Video conferencing in WeChat, which was used for real-time problem-based-learning discussion.

WeChat is an app available on Android, iPhone, BlackBerry, Windows, and Symbian platforms and is supported by Wi-Fi, 3G, and 4G data networks. Students can communicate with each other on WeChat at any time anywhere. This new mode of PBL was designed based on the WeChat app. The WeChat group worked like the traditional PBL group, comprising 6-8 students and a tutor (Figure 2). The PBL group members performed the WeChat-PBL by sending and receiving texts, images, voice messages, videos, and documents (eg, MS PowerPoint, MS Word, and Adobe PDF) (Figure 3). In addition, the PBL group members had real-time discussions using the video conferencing function in WeChat (Figure 4).

**Figure 5:**
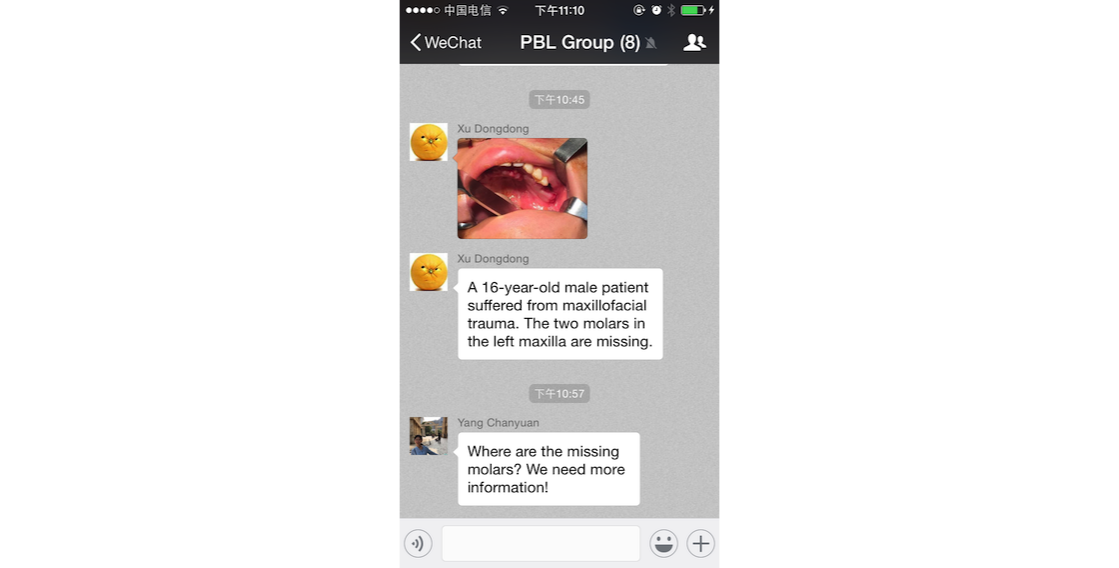
A real case was selected as the problem-based-learning (PBL) case by students, and the clinical data were uploaded to the WeChat-PBL group.

The dental students themselves selected the PBL cases. There were no definitive criteria to assess the PBL cases, and the number of the cases was not prespecified. Students selected the PBL cases, based on the clerkship intent and initially guided by the tutor. When a student saw a potentially suitable case in their clerkship, they would upload the clinical data of the case (including basic information, chief complaint, symptoms, signs, and laboratory examinations) to the WeChat-PBL group (Figure 5). The group members would discuss and decide whether the case was chosen for the PBL. The tutor then approved the case put forward by the students, aiming to ensure the quality of case.

**Figure 6:**
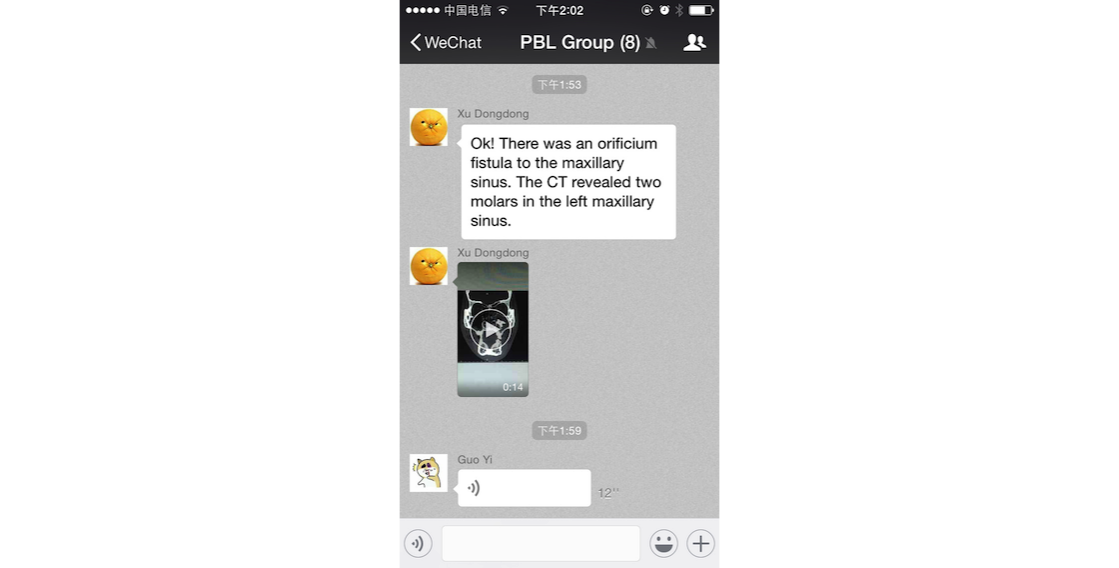
In the WeChat-PBL (problem-based-learning) group, the group members carry out the discussion by sending texts, images, voice messages, and videos.

**Figure 7:**
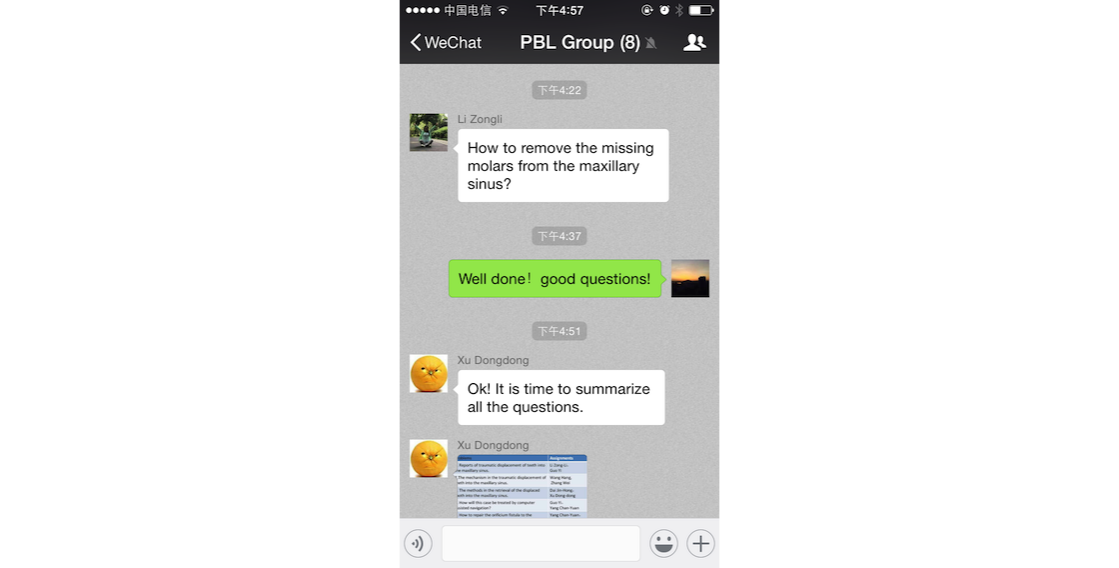
In the problem-based-learning discussion, questions were summarized and assigned to group members.

**Figure 8:**
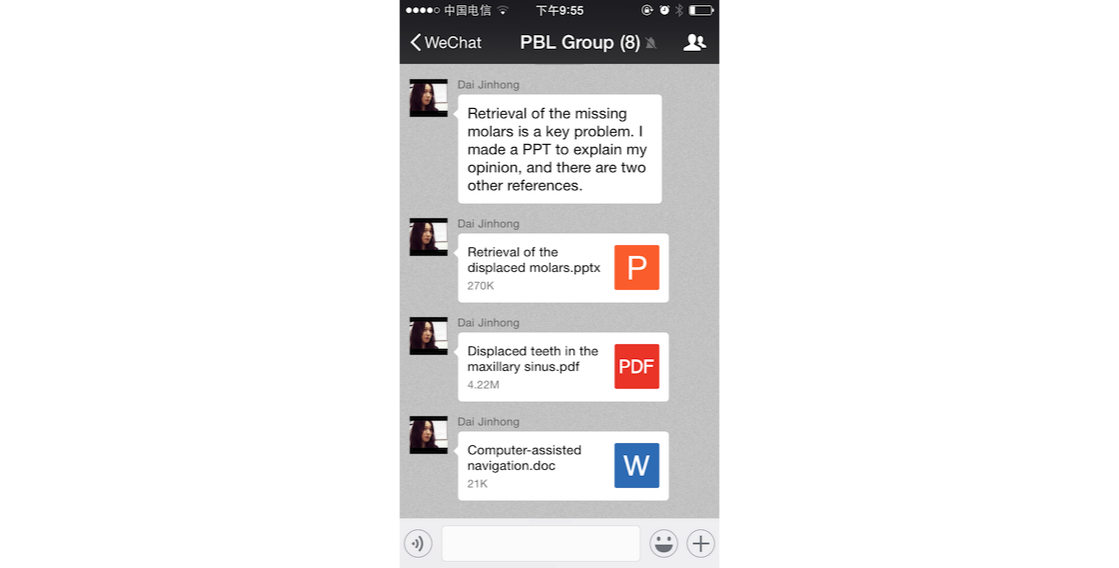
A group member expressed opinions on a question after literature review and learning, and then shared the relevant documents with group members.

**Figure 9:**
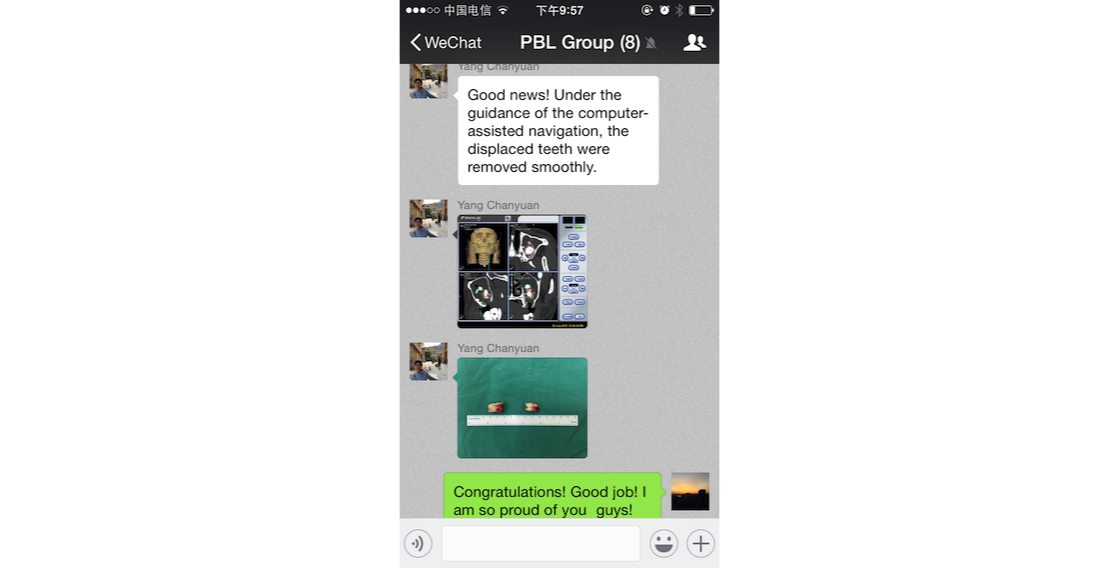
Problem-based-learning discussions align with the clinical treatment of the selected case.

Due to WeChat’s powerful functions, the group members could upload texts, images, voice messages, and videos easily and instantly (Figure 6). The group members asked questions about the selected case in the WeChat-PBL group, then discussed the various issues and summarized the questions within several days (Figure 7). The questions were then classified and assigned to the group members. Group members would then find the information, search for possible answers, and post their opinions by text, image, voice, video, or other forms (ie, documents) (Figure 8). Furthermore, the PBL discussion aligned with the clinical treatment of the selected case (Figure 9).

The tutor joined in the WeChat-PBL discussion and occasionally gave guidance to students. The tutor’s aim was to observe the performance and the number of active students in this new mode of PBL. The tutor was not supposed to answer the questions summarized by the students. The main duties of the tutor were as follows: (1) guide the students for deeper and wider thinking, and (2) ensure that every group member is participating in the discussion.

### Evaluation

The key to evaluating the WeChat-PBL was to infer whether the students were effectively gaining knowledge and improving skills, as well as developing independent clinical thinking and team skills. The evaluations included two sections: (1) periodic evaluation of the PBL process after each individual PBL case, and (2) long-term evaluation of the whole WeChat-PBL experience after the 3-month clerkship.

After each individual PBL case, the students and tutor were required to finish the periodic evaluation, including self-evaluation and peer evaluation, which comprised discussions in the WeChat group. The tutor also evaluated each student’s behavior and performance in the WeChat-PBL.

The long-term evaluation of the whole WeChat-PBL experience after the 3-month clerkship was summarized by a questionnaire survey to each student (Table 1). The questionnaire was designed and modified based on previous studies [14-16] and the objectives of this study. The questionnaire was validated by a pilot study with a smaller sample.

**Table 1:** Questionnaire results after 3 months of clerkship.

Questions	Yes, n (%)	No, n (%)
1. Did you enjoy discussing the PBL cases from the clerkship?	70 (97.22)	2 (2.78)
2. Did you enjoy communicating by WeChat in the online PBL?	72 (100)	0 (0.00)
3. Did you contribute to the group discussion?	60 (83.33)	12 (16.67)
4. Did you complete the tasks assigned to you on time?	58 (80.56)	14 (19.44)
5. Did you apply prior knowledge to solve problems?	62 (86.11)	10 (13.89)
6. Were you able to provide new information?	65 (90.28)	7 (9.72)
7. Did you feel free to propose questions?	70 (97.22)	2 (2.78)
8. Did you pay attention to the relevance of the information?	70 (97.22)	2 (2.78)
9. Did you assess the information you acquired critically?	59 (81.94)	13 (18.06)
10. Did you actively participate in interactive discussion?	60 (83.33)	12 (16.67)
11. Were you able to communicate ideas effectively with your group members?	61 (84.72)	11 (15.28)
12. Did your tutor consistently show enthusiasm with the PBL?	70 (97.22)	2 (2.78)
13. Did your group members and tutor offer feedback often to improve your learning?	69 (95.83)	3 (4.17)
14. Do you now have a better understanding of PBL?	72 (100)	0 (0.00)
15. Do you think WeChat is an effective app for online PBL in a dental clerkship?	72 (100)	0 (0.00)
16. Do you agree that online PBL based on WeChat can improve students’ ability in the clerkship?	71 (98.61)	1 (1.39)

### Analysis

The data from PBL cases and WeChat messages were collected for quantitative analysis in MS Excel 2013. The content of the periodic evaluations within the WeChat-PBL groups was analyzed qualitatively. The questionnaires for long-term evaluation were collected and analyzed, gathering participants’ opinions and comparing them, in order to understand whether the students benefited from this WeChat-PBL mode.

## Results

### Problem-Based Learning Cases

In this study, there were 45 cases presented in the WeChat-PBL within the 3-month clerkship, and each group finished four or five PBL cases. These 45 cases were all real clinical cases, including diseases from different departments (Oral & Maxillofacial Surgery, n=12; Endodontics, n=10; Periodontics Department, n=7; Prosthodontics, n=5; Orthodontics, n=5; Pediatric Dentistry, n=4; Dental Implantology, n=2). The multidisciplinary team approach was used in the discussion of 30 cases (66.67%). It took 15-22 days (mean 20.12 days) to finish one PBL case.

### WeChat Messages

All students had positive reactions to the communication within the groups. A total of 2063 text messages, 2325 voice messages, 364 images, 92 videos, 278 documents, and 129 webpage links were sent in the 45 PBL cases. In addition, there were 54 video conferences. Of the 2063 text messages, 1823 came from students and 240 from tutors. Of the 2325 voice messages, 2068 were from students and 257 from tutors. All of the images, videos, documents, and webpage links were sent by students.

### Periodic Evaluation

The periodic evaluation showed that students and tutors were quite satisfied with the process of WeChat-PBL and appreciated the group members’ performance. The students held positive attitudes toward the WeChat-PBL teaching, noting that WeChat-PBL encouraged the activation of prior knowledge and provided opportunities for elaboration on that knowledge. The tutors reported that the students had obvious increases in both general knowledge and the skills for analyzing problems.

### Long-Term Evaluation

The response rate to the questionnaire was 100% (72/72). The results of the questionnaire indicated that the WeChat-PBL achieved positive effects (Table 1). All the students commented positively about communicating on WeChat in the online PBL. Most students enjoyed discussing the real PBL cases from the clerkship (70/72, 97.2%), felt free to propose questions (70/72, 97.2%), and paid attention to the relevance of the information (70/72, 97.2%). As well, most students reported that they were able to provide new information (65/72, 90.28%) and the group members and tutor often offered feedback to improve their learning (69/72, 95.8%).

In addition, most of the students evaluated themselves highly on other aspects in the PBL, including contributing to the group discussion (60/72, 83.3%), completing the assigned tasks on time (58/72, 80.5%), applying prior knowledge to solve problems (62/72, 86.1%), assessing the acquired data critically (59/72, 81.9%), participating in the interactive discussion actively (60/72, 83.3%), and communicating ideas effectively with group members (61/72, 84.7%).

The majority of the students (70/72, 97.2%) felt the tutors showed persistent enthusiasm in the WeChat-PBL, and the tutors gave a positive evaluation of all the students’ behavior in the PBL. All students reported having a better understanding of PBL and indicated that WeChat was an ideal app for online PBL in the dental practical clerkship. Similarly, almost all students agreed that the WeChat-PBL could improve students’ skills in the clerkship.

## Discussion

### Principal Results

In this study, we designed a new PBL mode based on WeChat. This mode of online PBL successfully eliminated the physical and temporal limitations of traditional PBL in dental clerkships. It ensured the time needed for and the quality of PBL, broadened the manner that students gain knowledge, and promoted efficiency in solving problems in the dental practical clerkship. As a modern pedagogical philosophy, the importance of PBL is increasingly being recognized in student learning and innovation in medical education [17]. Many educators have tried to improve traditional PBL by modifying the instruction, hence, other PBL modes such as tutorless PBL, 3C3R Modified PBL, and Hybrid-PBL have emerged in PBL teaching [18-20]. However, compared with traditional PBL, the WeChat-PBL has several advantages that take PBL to a higher level.

The most important advantage of our WeChat-based PBL is its timesaving and convenience. Traditional PBL is time-consuming [21], since a regular schedule is needed to gather the students together. However, WeChat-PBL allows asynchronous discussions and supports discussions “anytime, anywhere.” Furthermore, group members can upload images, videos, radiological examinations, or laboratory results of the case via mobile phone in a timely manner, so that other members can instantly access the case data.

Second, the new PBL mode offers an excellent platform for sharing educational resources and the newest information. Group members can use their mobile phones, searching valuable information on the Internet. Many universities offer a range of online e-Health courses and other online curricula [22,23]. Group members can send webpage links to these and other resources to the group chat directly, thus, improve the efficiency and quality of the discussions in the PBL.

Third, this new mode improves students’ motivation to learn and strengthens their practical ability. The questionnaire results indicated that the students became more and more curious and active in WeChat-PBL, aiming to grasp the practical use of theoretical knowledge instead of just knowing about the theory. Group members became better at listening and communicating in the new PBL mode, which helped them achieve the ultimate intent of the clerkship.

Social media has been gaining steady support for its presence in medical education. The findings of this study illustrate a method through which online PBL teaching can be facilitated by the use of social networking sites. Compared with the use of similar social media platforms such as WhatsApp [24] and Twitter [25] in PBL, WeChat has some special functions that are particularly well adapted for online PBL discussions including video conferencing and sharing of documents (eg, MS PowerPoint, MS Word, and Adobe PDF). Another noteworthy feature of WeChat-PBL in our study is that the PBL case was directly selected from students’ clinical work, and the PBL discussions aligned with the clinical treatment of the selected case. These real cases helped students recognize the authenticity of the study materials and become more motivated to explore and solve the problems [26]. Moreover, the students were able verify the solutions in the practice, truly integrating theory with practice. In this study, each PBL group consisted of students belonging to more than one department, and the teachers were randomly assigned across departments. These features contribute to the students’ dental practice in different clinical departments in their clerkship rotation, integrating the knowledge of different disciplines in dentistry. Further, the tutor approved the case selected by the students instead of preparing a case, allowing the tutor more time and energy to focus on the guidance.

### Limitations

In order to use this new PBL mode based on WeChat, there are several basic requirements: a suitable mobile phone, the WeChat app, and Internet or Wi-Fi access. For students in China, owning a mobile phone is not a financial burden. In this study, all students had their own phones. Furthermore, WeChat is a free app that anyone can download and install on a supported mobile phone. These days, all colleges and universities in China provide a Web-based information platform for their campuses. Free wireless networks are ubiquitous in student dormitories and in hospitals. However, there exist several shortcomings in this new PBL mode.

First, the dental students have more freedom using WeChat-PBL than in traditional PBL. This helps motivate students to spend more time in the PBL. When there is increased control in traditional PBL, students’ motivation to assume responsibility for learning decreases [27]. However, too much freedom can lead to laziness, procrastination, and so on. Without the restrictions of the classroom setting, some undisciplined students may send off-topic pictures or messages to amuse others in the PBL group chat. Quality pedagogy needs to balance freedom and restraint [28]. Therefore, the PBL group members should set and enforce guidelines and rules in the group chat. If necessary, the tutor could warn the undisciplined students privately. Fortunately, this phenomenon never occurred in this study. Students were motivated and stayed on topic.

Second, this online PBL mode lacks ways to manage discussion topics effectively. The personality types of students and their group dynamics are linked to their PBL performance [29]. During an online discussion, such as the WeChat-PBL, the topics can be dominated by a few active users, since there is not the restriction of traditional classroom setting. Students can discuss what they are interested in and can go off topic unintentionally, potentially affecting the enthusiasm and activity of the other students. Keeping students engaged in a virtual environment requires a sustained instructor presence. In order to overcome this shortcoming, the tutor should always be active in the asynchronous discussion group, encouraging students’ discussion and prompting them to think more deeply. In addition, students admitted to the WeChat-PBL should receive training on this new mode as part of the PBL orientation program. In our study, we found that all students were able to focus on the discussion topics after receiving training.

As far as the study method is concerned, there were some limitations to our design. The study lacked a control group, and the evaluations focused only on perception/satisfaction with the PBL, rather than assessing actual changes in knowledge or skills. In addition, the tool used to evaluate the experience was not validated, and thus the satisfaction reported may be due to the novelty effect. Therefore, longer studies are needed to assess intermediate and long-term effects. Finally, the study lacked a rigorous sampling process, and all eligible students were recruited into our study.

### Conclusions

The results indicate the feasibility and acceptability of WeChat in PBL teaching for students in a dental practical clerkship. This new PBL mode not only has the advantages of timesaving and convenience but also offers a suitable platform for sharing educational resources and the newest information. The WeChat-PBL improves students’ learning motivation and strengthens their practical ability. It also contributes to students’ dental practice in different clinical departments in their clerkship rotation, integrating the knowledge of different disciplines in dentistry. Although there are certain limitations in WeChat-PBL, we were able to find the solutions during the study process. We conclude from this study that our WeChat-PBL is an effective method for PBL teaching in a dental practical clerkship.

## Abbreviations

PBL: problem-based learning
